# How Is Working Memory Related to Reading Comprehension in Italian Monolingual and Bilingual Children?

**DOI:** 10.3390/brainsci13010058

**Published:** 2022-12-28

**Authors:** Margherita Orsolini, Francesca Federico, Michele Vecchione, Giorgia Pinna, Micaela Capobianco, Sergio Melogno

**Affiliations:** 1 Department of Psychology of Development and Socialization Processes, Faculty of Medicine and Psychology, Sapienza University of Rome, 00185 Roma, Italy; 2 Faculty of Psychology, International Telematic University Uninettuno, 00186 Rome, Italy; 3 Faculty of Psychology, “Niccolò Cusano” University of Rome, 00166 Rome, Italy

**Keywords:** reading comprehension, working memory, episodic buffer, bilingualism

## Abstract

This study explored how working memory resources contributed to reading comprehension using tasks that focused on maintenance of verbal information in the phonological store, the interaction between the central executive and the phonological store (WMI), and the storage of bound semantic content in the episodic buffer (immediate narrative memory). We analysed how performance in these tasks was related to text decoding (reading speed and accuracy), listening and reading comprehension. The participants were 62 monolingual and 36 bilingual children (mean age nine years, SD = 9 months) enrolled in the same Italian primary school. Bilingual children were born to immigrant parents and had a long history of exposure to Italian as a second language. The regression analyses showed that reading accuracy and listening comprehension were associated with reading comprehension for monolingual and bilingual children. Two working memory components—WMI and immediate narrative memory—exhibited indirect effects on reading comprehension through reading accuracy and listening comprehension, respectively. Such effects occurred only for monolingual children. We discuss the implications of such findings for text reading and comprehension in monolinguals and bilinguals.

## 1. Introduction

Children born to immigrant parents and attending grades 2 and 5 in Italian schools in 2014–2015 [1] and 2019 [2] showed lower performance in reading comprehension than their monolingual peers in a large representative sample of schools throughout the country. As reading comprehension is a crucial factor affecting educational outcomes in most school subjects, understanding the factors underlying such disparity between first- and second-language learners is essential for developmental psychology.

### 1.1. The Processes Involved in Text Comprehension

Text comprehension is like a mental weaving: semantic structures derived from reading are integrated with the reader’s background knowledge and interlaced with inferential links to form the thread of a mental representation that is updated as the reading proceeds [3,4]. Although such an incremental process is conceived as relatively passive and automatic [5], the reader’s intentional and controlled actions also constrain reading comprehension, allowing awareness of comprehension gaps, monitoring the local semantic congruence (e.g., the reader rereads a sentence or checks for a word meaning), enhancing a text’s elaboration (e.g., the reader underlines or writes a note). While children’s use of intentional and controlled actions has rarely been the focus of developmental research (see [6]), many studies have analysed the influence of language skills and working memory on monolingual and bilingual children’s reading comprehension.

### 1.2. Oral Language and Reading Comprehension in Monolinguals and Bilinguals

The main prediction of the well-known *simple view of the reading* model [7,8] is that reading comprehension depends on decoding skills and oral language comprehension: different combinations of efficiency in such partly independent processes explain children’s text comprehension levels. Such a prediction has been confirmed by many studies conducted in several languages [9,10,11,12]. In languages with irregular orthographies, the influence of decoding on reading comprehension decreases with development [13,14], whereas the impact of listening comprehension increases with more advanced decoding skills [15,16]. On the contrary, for languages with regular orthographies, listening comprehension is a strong predictor of reading comprehension, even in the early stages of reading [12,17,18].

Focusing on bilingual children who are regularly exposed to two or more languages —one of which is a minority language in the country in which they live—and comparing their reading development to monolingual children, the meta-analytic review of Melby-Lervåg and Lervåg [19] found a significant mean size effect in favour of the monolinguals’ reading comprehension. The gap between monolingual and bilingual groups was smaller in Canada than in Europe and the United States for reasons probably linked to the immigrants’ higher levels of instruction in Canada. Despite their different performance levels, written text comprehension was closely related to oral language and decoding skills for both monolinguals and bilinguals.

As the studies considered by the meta-analytic review of Melby-Lervåg and Lervåg [19] measured decoding in different ways, analysing isolated word recognition, reading word list, or accuracy and fluency in passage reading, it is not clear which decoding component (e.g., accuracy or reading speed) is mainly involved in reading comprehension.

In summary, using listening comprehension as a proxy for oral language skills, several studies found that this factor, along with decoding skills, was a predictor of reading comprehension in monolingual and bilingual children.

### 1.3. Oral Language and Decoding Skills Predicting Differences between Monolingual and Bilingual Children in Reading Comprehension

The results of the Melby-Lervåg and Lervåg [19] meta-analysis, in line with other studies [20,21,22,23], pointed to L2 listening comprehension and its underlying language skills (e.g., vocabulary or sentence comprehension) as the main factors predicting differences in reading comprehension between bilinguals and monolinguals. Decoding skills, which demonstrate a small but significant difference between monolinguals and bilinguals [19], decrease their influence as children age.

The influence of listening comprehension on reading comprehension in bilingual children is moderated by prolonged exposure to the L2 language. Comparing early and late bilinguals—children who learned L2 before or after four years of age—to monolingual controls, Bonifacci and Tobia [24] found that reading and listening comprehension were at the same level as controls for early bilinguals but at a lower level for late bilinguals. Weaker listening comprehension and decoding skills were the factors underlying lower reading comprehension in late bilinguals.

Differences in listening and reading comprehension between monolingual and bilingual children also seem to be moderated by socio-economic status: Melby-Lervåg and Lervåg [19] found more significant differences in oral language comprehension between monolinguals and bilinguals from a low-SES home. Bonifacci, Lombardo, Pedrinazzi, Terracina and Palladino [25], on the other hand, observed that bilinguals with low SES did not show significantly weaker listening comprehension than monolinguals with low SES but lower levels of written text comprehension than both high- and low-SES monolinguals.

### 1.4. Working Memory and Reading Comprehension in Monolinguals and Bilinguals

According to Baddeley’s influential model [26,27,28], working memory consists of a central executive whose limited capacity for attentional control is responsible for the active maintenance and processing of task-relevant information, which is temporarily held in domain-specific verbal and visuospatial stores or a multi-modal episodic buffer [26,28].

Investigating working memory with tasks such as the backward digit recall, letter/number sequencing or the operation span, some studies found that the executive components of WM explain a significant portion of the variance in reading comprehension both in typically developing children [29,30,31] and in those with poor comprehension [32]. Thus, reading processes asking for more complex and active linguistic elaboration involve verbal WM tasks tapping the central executive.

Other studies found, however, that decoding (e.g., word recognition) and text comprehension are related to the executive components of WM to a similar degree [33,34,35], suggesting that word reading may continue to require an active integration of information (e.g., phonological, orthographic and semantic components) that involves WM resources.

We may ask whether WM is related to oral and written text comprehension with direct effects or whether such a relationship is mediated by vocabulary and decoding skills. A meta-analytic review [34] found that WM was related to listening and reading comprehension with similar size effects. Such effects, however, were primarily indirect: when decoding and vocabulary were controlled, the WM’s direct effect on written or listening comprehension disappeared. A different finding emerged, however, from a study analysing the longitudinal contribution of decoding, language comprehension, WM and other executive functions [36]. In this study, the children’s reading comprehension was longitudinally predicted by WM with both a direct and an indirect effect (via decoding skills).

The study by Nouwens, Groen and Verhoeven [33] suggests that WM may enhance reading comprehension by facilitating semantic processing and storage. The task used in this study consists of a category-cued recall, in which the participants read a list of nine words and are asked at the end to recall the nouns according to a specific category, such as *fruit*. As such a task requires semantic and conceptual elaboration, it needs to be clarified whether it involves storage or executive components of WM. How semantic content storage affects listening and reading comprehension is a relevant question that has to be further explored.

The contribution of WM to reading development in bilingual children has received a less systematic investigation. Swanson, Orosco and Kudo [35] involved a large sample of grade 1–3 Hispanic children in a longitudinal study in the southwest United States and found that reading growth in decoding (word identification) and written text comprehension was related to the executive components of WM. The executive component of WM also predicted the reading growth parameters in children who turned out to develop a reading disability, and this confirmed the significant involvement of WM in L2 reading performance found in previous studies [37,38].

When WM is considered along with oral language skills, its indirect contribution to reading comprehension via language skills (e.g., vocabulary or syntactic skills) has been observed for both monolingual and bilingual children [39].

### 1.5. The Study’s Aims

Our study explored the relationships between reading comprehension and WM in monolingual children who were only exposed to Italian at home and bilingual children who had one or both parents who immigrated to Italy from other countries, were regularly exposed to one minority language and have had a long history of exposure to Italian.

The literature overview in the previous sections suggests that WM can influence the two main predictors of reading comprehension: listening comprehension and word decoding. However, it needs to be clarified which components of these two predictors are related to WM. Regarding decoding, we will ask in this study whether an interaction between the central executive and the phonological store enhances the correctness of word recognition or reading speed when children read a text. For listening comprehension, the role of semantic content storage [33] needs to be clarified. This study will explore whether the episodic buffer component of WM—which, in Baddeley’s model, allows semantic content to be bound through long-term knowledge [28,40]- is involved in listening comprehension.

Our study, after an analysis of the role of WM in decoding and listening comprehension, addresses two main issues, asking whether WM resources influence written text comprehension through a direct or an indirect influence, which is mediated by decoding skills and listening comprehension, and whether the involvement of WM in reading comprehension is similar for monolingual and bilingual children.

## 2. Materials and Methods

### 2.1. Procedure

Approval for this study was obtained from the Ethics Committee of the Department of Developmental and Social Psychology, Sapienza University of Rome. The first author contacted schools with a high enrolment of bilingual students. Contact was established over the phone. One school interested in participating and located in a suburban area of Rome was selected. The school’s principal, the children’s teachers and parents or guardians were informed with a letter detailing the project’s aims and procedures. All the children in grades 3, 4 and 5 received parental consent and participated in the assessment sessions. Every child was individually tested in 4 sessions lasting about 50 min each, with tasks assessing attention, processing speed, non-verbal reasoning, working memory, language and reading. The current study will analyse only some of the tasks’ results. Each participant also completed a questionnaire in groups of 4–5 children. All the assessment sessions took place inside the school building, in two special classrooms reserved for the project, on days and times indicated by the teachers. If the child showed impatience or no longer wished to continue, the meeting was suspended and resumed another day. The teachers played an intermediary role between parents and examiners in delivering a parents’ questionnaire completed at home. All questionnaires were returned in a sealed envelope to guarantee privacy and anonymity. Data collection began in November 2019 and ended in early March 2020 due to the spread of COVID-19. For this reason, some children could not complete some tests and were excluded from the study.

### 2.2. Participants

The final sample consisted of 98 children (boys = 61.22%; girls = 38.78%; mean age = 113.55 months, SD = 9.15, range= 95.4–136.40 months) attending grades 3, 4 and 5. The inclusion criteria for the analyses carried out in this study were that the child had not received any diagnosis for neurodevelopmental disorders and had a score higher than 80 in the Raven’s Coloured Progressive Matrices test.

The sample had 62 monolingual (66.13% boys, 33.87% girls; mean age = 113.09 months, SD = 9.01, range = 97.74–136.40 months) and 36 bilingual children (52.78% boys, 47.22% girls; mean age = 114 months, SD = 9.48, range = 95.44–132.09). The two groups did not differ for nonverbal reasoning, as expressed by z scores on the Raven’s Coloured Progressive Matrices (t(98) = 0.63, *p* < 0.52, d = 0.12).

The monolingual children were only exposed to the Italian language at home. In the few cases in which one or both parents were not Italian native speakers, they had resided in Italy for at least ten years and reported that their dominant language at home was Italian that their comprehension and production of Italian was excellent; their questionnaire stated that the child had no or very low understanding of the parent’s L1.

The bilingual children had been exposed to an L1 different from Italian from birth and Italian either from birth or before age 4. We included in this study only children whose parents reported that the child’s comprehension of L1 was good or excellent.

### 2.3. Participants’ Socio-Cultural and Language Characteristics

The participants lived in a suburban area of Rome characterised by low or medium-low SES (socio-economic status). Most children’s parents had a medium-low level of education with the prevalence of a secondary school qualification (fathers 39.8%; mothers 41.9%) or a middle school certificate (fathers 34.7%; mothers 28.6%). Few parents had a college degree (fathers 7.1%; mothers 9.2%). We measured the parents’ level of education as the number of years at school after five years of age. We added the years of education of the two parents in the analyses that controlled the influence of the parents’ education on children’s reading comprehension. The number of years at school doubled for our sample’s seven single-mother or father families. Monolingual and bilingual children did not differ significantly in terms of their parents’ level of education (t(98) = −0.99, *p* < 0.32, d = 0.20)).

Most bilingual children were born in Italy (89%), and the remainder had settled in Italy within their first three years of age. The L1 languages of bilingual children included Romanian (41.7%), Spanish (19.5%), Sinhalese (13.9%), Polish (5.6%), Moldovan (5.6%), Albanian (5.6%) and other (8.4%).

### 2.4. Text Reading and Comprehension, Listening Comprehension

Text reading, listening and reading comprehension were assessed with the ALCE battery [41] (Reliability: Cronbach’s alpha = 0.74 to 0.83). Children were asked to read a narrative passage and informed that at the end of their reading, they would receive some questions to answer. Text reading speed was analysed in syllables per second, and the total number of errors was recorded for a reading accuracy score. Reading comprehension was assessed with ten open questions presented orally, whose answers received 0–2 points score (maximum score: 20).

For listening comprehension, the experimenter first read a narrative passage aloud and then asked ten open oral questions scored with the same method as the one used for reading comprehension (maximum score: 20). For both reading and listening comprehension, five of the questions tagged information explicitly presented in the passages (local comprehension). The other five questions involved inferential reasoning (global comprehension).

The raw scores were transformed into a T standard score using the battery’s normative data.

### 2.5. Assessment of Working Memory

#### 2.5.1. Non-Word Repetition

We assumed that non-word repetition—a task that taps into the working memory’s phonological store [42]—is involved in phonological reading. Several studies have shown that non-word repetition predicts reading performance in typical and atypical reading development (see [43,44]).

We used a task which is a part of the Italian VAUMELF battery [45] (Reliability: Cronbach’s alpha = 0.90 to 0.99). The test consists of 40 pseudo-words presented via an audio track with an interval of 5 s. The child was asked to listen to each pseudo-word and immediately repeat it. Each child’s repetition received a scored of one if the pseudo-word was correctly repeated. The final raw score (maximum score: 40) was transformed into a zeta score considering the battery’s normative data.

#### 2.5.2. The Working Memory Index (Wmi)

We analysed the interaction between the central executive and the phonological store using the WISC IV Working Memory Index (WMI), involving both maintenance (direct digit span) and elaboration (backward digit span and the number/letter sequencing) working memory processes. We considered WMI to measure the effective interaction between the central executive and the phonological store. Three subtests from the Italian version of the fourth edition of the Wechsler Intelligence Scale for Children [46] (Reliability: Cronbach’s alpha = 0.87 to 0.92) contributed to the working memory index (WMI): Digit Span (forward and backward) and Letter-Number Sequencing. Forward Digit Span requires the child to repeat numbers in the same order as read aloud by the examiner. Backward Digit Span requires the numbers to be repeated in the reverse order of that presented by the examiner. The test is interrupted if the child incorrectly repeats two sequences of digits of the same item. In Letter-number sequencing, the child is asked to repeat a series of numbers and letters in ascending and alphabetic order (e.g., 4-B-1-A→1-4-A-B). The task is interrupted when a child obtains a score of 0 on three items. Using the WISC IV Italian normative data [47], the children’s raw scores on (a) direct and backward digit span and (b) letter/number sequencing were first converted into scaled scores, and then their sum was converted into a QI score.

#### 2.5.3. Immediate Narrative Memory

The relationship between the episodic buffer and text comprehension has been analysed in this study through an immediate narrative memory task. Although other types of verbal tasks might be used [48], we chose immediate recall of a short oral narrative passage to tap a passive temporary storage system in which verbal-semantic information is integrated thanks to the contribution of long-term knowledge [26,40]. In the task drawn from the Nepsy II battery [49] (Reliability: Cronbach’s alpha = 0.69 to 0.71), the child was first asked to listen to a short story and then to recall it immediately afterwards (free recall score: 0–20). Credit was given for each story element retrieved correctly, irrespective of whether the recall was verbatim, expressed with a similar meaning or in a different sequence from the original story. The test also required the child to answer open questions about the details that were not spontaneously retrieved (cued recall) and eventually answer closed questions (recognition score); only the free recall score was used in the analyses of our study. This score—which is related to immediate memory retrieval—was converted to a scaled score (mean = 10, st.dev. = 3) using Nepsy II’s Italian normative data [50].

### 2.6. The Parent’s and Child’s Questionnaire

The parents’ version of the questionnaire *Languages, Discourses and Reading*, gives details about the child’s birth, the family composition, the parents’ employment and educational qualifications, their country of origin and years of permanence in Italy, the languages spoken at home, the frequency of discourse activities carried out with the child and both the child’s and parents’ proficiency in L1 and L2 (this part was only reserved for parents whose L1 was not Italian).

The children’s questionnaire gathered information on the use of languages in the family, at school and with friends or classmates. The experimenters supervised the compilation and answered the children’s requests for clarification.

### 2.7. Statistical Analysis

Independent sample t-tests were run to compare monolingual and bilingual children’s decoding skills, oral and written text comprehension and working memory components. A correlation analysis explored the involvement of the different WM components with decoding by distinguishing reading accuracy and speed. The same analysis clarified whether the episodic buffer—with its function of semantic content storage—is related to listening and reading comprehension.

Multiple regression analyses were run to test whether two main components of WM—the interaction between the executive and the phonological store (WMI) and the episodic buffer (Immediate Narrative Memory)—have a direct or indirect relationship with reading comprehension. Considering our correlation results, we chose two mediator variables -listening comprehension and reading accuracy—and explored whether WM (the WMI and Immediate Narrative Memory) affected such two mediators and, through them, exerted an indirect effect on reading comprehension. The same multiple regression analyses also tested whether the involvement of working memory in reading comprehension differed in monolingual and bilingual children. We thus examined whether the indirect effect of WM on reading comprehension—through listening comprehension and reading accuracy —was moderated by linguistic status.

The analyses have been performed using Model 7 of the SPSS macro-PROCESS [51]. The model allows a single independent variable with multiple mediators. We, therefore, performed two separate regressions, using WMI (Model 1) (Supplementary Materials Table S1) and immediate narrative memory (Model 2) (Supplementary Materials Table S2) as predictors, listening comprehension and text reading accuracy as mediators, and reading comprehension as the criterion, respectively. Linguistic status (coded 0 = monolingual and 1 = bilingual) was posited as moderating variable. Parameters were estimated after controlling for parents’ years of education, which was included as a covariate. Indirect effects were tested for significance using 95% bias-corrected confidence intervals based on a bootstrapping procedure with 5000 replications.

## 3. Results

### 3.1. Do Monolingual and Bilingual Children Show Different Performance Trends in WM Measures, Decoding, Listening and Reading Comprehension?

Table 1 shows the descriptive statistics for the measures included in this study. The data approximated a normal distribution for each measure, but children’s performance was low in the non-word repetition test.

We first analysed whether children in the monolingual or bilingual groups scored differently on the working memory and reading measures. To this end, we performed a series of independent samples *t*-tests, adjusting for multiple comparisons through a Bonferroni–Holm correction [52]. After this adjustment, results showed no significant differences in all examined variables (see Table 1). As reported in Table 1, the results showed no significant differences in all examined variables. The effect size was mainly in the small range (Cohen’s d < 0.50).

### 3.2. Exploring the Relationship between WM Components and the Two Main Predictors of Reading Comprehension (I.E. Decoding and Listening Comprehension) in Monolingual and Bilingual Children

Table 2 shows the correlations for the monolingual (below the diagonal) and bilingual (above the diagonal) groups between all the measures used in the study. For both monolinguals and bilinguals, reading comprehension was positively correlated with reading accuracy and listening comprehension.

For monolinguals, non-word repetition was positively correlated with reading speed and accuracy, whereas for bilinguals, there was a significant correlation only with reading speed. The working memory index was positively correlated with reading accuracy only for monolinguals, whereas it was positively correlated with reading speed and reading comprehension only for bilinguals.

Listening comprehension positively correlated with immediate narrative memory for monolinguals, whereas it was positively correlated with non-word repetition and reading speed for bilinguals.

### 3.3. Does the Interaction between the Central Executive and the Phonological Store (WMI) Influence Reading Comprehension Similarly for Monolingual and Bilingual Children?

**Figure 1:**
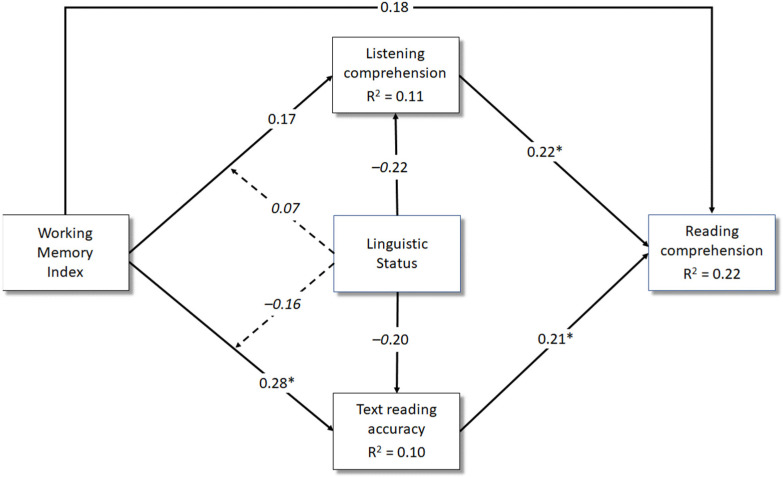
Model 1: A multiple regression analysis including the working memory index (predictor), listening comprehension and reading accuracy (mediators), linguistic status (moderator) and reading comprehension (outcome). *Notes*. * *p* < 0.05. Dashed lines indicate the moderating effect of linguistic status. Parents’ years of education were not represented to avoid cluttering the figure. Standardised betas for this covariate were 0.19 (*p* = 0.07) for listening comprehension, 0.12 (*p* = 0.21) for text reading accuracy and 0.10 (*p* = 0.31) for reading comprehension.

Figure 1 reports the results (standardised regression coefficients and R-squared) of Model 1 (with WMI as the predictor). As can be observed, the working memory index (WMI) significantly and positively predicted word reading accuracy, while it did not relate to listening comprehension. These associations did not differ across mono- and bilingual children (i.e., no statistical significance for the moderating role of linguistic status). Both reading accuracy and listening comprehension, in turn, had a positive effect on reading comprehension. Finally, WMI had no direct effect on reading comprehension, though it exerted a significant indirect effect on reading comprehension via word reading accuracy. This effect, however, was observed among monolingual children (*β* = 0.06, 95% CI: 0.014, 0.134), but was not significant for bilinguals (*β* = 0.03, 95% CI: −0.029, 0.129). The indirect effect from WMI to listening comprehension was not significant in both groups (monolinguals: *β* = 0.04, 95% CI: −0.015, 0.124; bilinguals: *β* = 0.05, 95% CI: −0.015, 0.190).

### 3.4. Does the Episodic Buffer (Immediate Narrative Memory) Influence Reading Comprehension Similarly for Monolingual and Bilingual Children?

**Figure 2:**
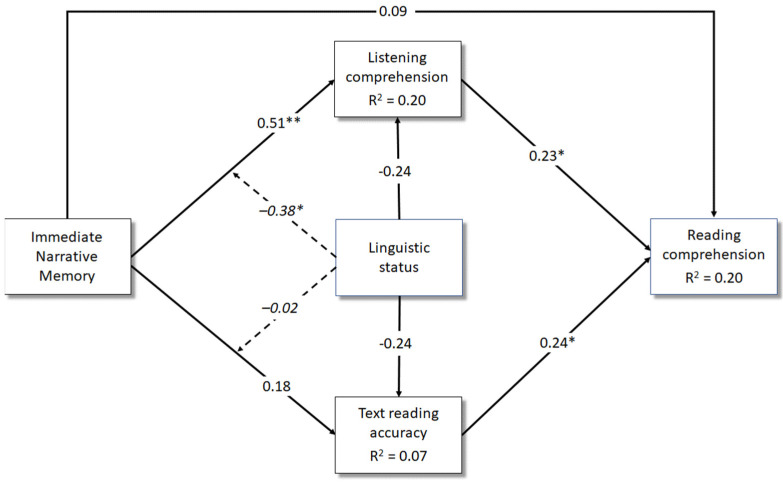
Model 2: A multiple regression analysis including immediate narrative memory (predictor), listening comprehension and reading accuracy (mediators), linguistic status (moderator) and reading comprehension (outcome). *Notes*. * *p* < 0.05; ** *p* < 0.01. Dashed lines indicate the moderating effect of linguistic status. Parents’ years of education were not represented to avoid cluttering the figure. Standardised betas for this covariate were 0.14 (*p* = 0.10) for listening comprehension, 0.13 (*p* = 0.22) for text reading accuracy and 0.11 (*p* = 0.28) for reading comprehension.

The results of Model 2 (immediate narrative memory as predictor) are shown in Figure 2. Immediate narrative memory exerted a positive effect on listening comprehension. This association, however, was found only among monolingual children, as revealed by a significant moderating effect of linguistic status (monolinguals: *β* = 0.51, *p* <0.001; bilinguals: *β* = 0.13, *p* = 0.39). The association with reading accuracy was not significant. Immediate narrative memory affected reading comprehension only indirectly through listening comprehension. Again, the indirect effect was significant only for monolingual children (monolinguals: *β* = 0.12, 95% CI: 0.005, 0.236; bilinguals: *β* = 0.03, 95% CI: −0.042, 0.142). In Models 1 and 2, parents’ years of education did not contribute significantly to any of the examined variables.

## 4. Discussion

This study found that bilingual children with a long history of exposure to Italian showed slightly lower reading comprehension than monolinguals. Although the difference did not turn out to be statistically significant, the trend was in line with the findings of several previous studies [19,20,21,25,53]. Bilinguals showed similar performance as monolinguals in decoding skills (i.e., text reading speed and word accuracy), listening comprehension, non-word repetition, immediate narrative memory and working memory tasks.

### 4.1. How Is Working Memory Related to Reading Decoding?

Focusing on the phonological store, tapped by the non-word repetition task, we found that such WM component was related to reading speed: the more children could store and repeat short and long pseudo-words, the more their text reading was quick (see correlations of Table 2). This finding suggests that reading speed is still affected -in Italian children who attend grades 3–5—by the fluency of phonemic blending that the phonological store supports. Reading accuracy of monolingual children involved both non-word repetition and WMI. As this latter measure tagged the interaction between the central executive and the phonological store in our study, our finding suggests that word recognition in text reading requires an active/controlled elaboration likely to involve lexical-semantic selection. Unlike monolingual children, the correlation between reading accuracy and WMI was weak and did not approach statistical significance for bilingual children. This finding is consistent with the study of Bellocchi, Tobia and Bonifacci [18], who found that reading accuracy was only associated with pseudo-word repetition in bilinguals. In contrast, it also involved lexical knowledge for monolinguals.

Conversely, in our study, reading speed correlated with WMI for bilingual children, suggesting that phonological reading, and its phonemic blending process, still rely on some active elaboration of the words’ orthographic and phonological structure.

### 4.2. Indirect Working Memory Contribution to Reading Comprehension

In line with earlier studies [12,13,14,15,16,17,18,19,20,21], word reading accuracy directly influenced reading comprehension for both monolinguals and bilinguals, as shown by the results of our regression models. However, for monolinguals, there was also an indirect WM effect, via reading accuracy, on reading comprehension. Thus, reading comprehension seemed to benefit from more active/controlled word recognition in monolinguals.

In line with earlier studies [12,21,24,25,54], we found that listening comprehension directly affected reading comprehension for both monolinguals and bilinguals. As listening comprehension is closely linked to lexical and syntactic skills [11,20], our findings suggest that oral language comprehension is the main factor underlying reading comprehension, as predicted by the *simple view reading* model [7,8].

We asked in this study whether listening comprehension was related to the episodic buffer component of WM and its semantic content storage function. Following Baddeley [40], we considered that temporary storage of narrative content in the episodic buffer depends on a relatively passive chunking process produced by activating long-term semantic knowledge. Such knowledge provides an incremental sequential binding of the narrative content that is enriched as listening proceeds and allows an integrated representation enabling comprehension [3,55].

We found that only the performance of monolingual children in the immediate narrative memory task—a measure of the episodic buffer in our study—was significantly associated with listening comprehension and indirectly contributed to reading comprehension, as shown by regression model 2. An explanation for the lack of such indirect influence of immediate narrative memory on bilinguals’ listening comprehension is that bilingual children may have used different strategies in the two tasks. During listening comprehension, in which children were aware that they should answer some final questions, they might have engaged in the long-term encoding of the story sentences [56]. In contrast, in the immediate narrative memory task, in which a request of spontaneous recall followed listening, they might have involved the phonological store in engaging in a covert rehearsal, as suggested by the high correlation with non-word repetition.

## 5. Conclusions

Our findings on monolingual children align with previous studies [33,34,54], suggesting that WM only indirectly facilitates reading comprehension. The WM’s activity underlying written text comprehension is often described as a complex dual process. Relevant semantic content is kept active in short-term memory while, at the same time, different sources of information are coordinated to constrain the building of a text representation. Such a dual process would require the direct involvement of the central executive in reading comprehension, which does not seem to occur in primary school children. The controlled process directly enhanced by WM in primary school children is not reading comprehension but decoding accuracy.

In our study, monolingual children’s comprehension of an oral text was strongly associated with WM’s capacity to temporarily bind and store narrative semantic content. In turn, such binding-storage function indirectly contributed to reading comprehension via listening comprehension. Thus, the storage of bound semantic content, in line with the conclusion of Nouwens, Groen and Verhoeven [33], is another contribution that WM offers to children’s reading comprehension.

We also asked whether WM resources were used differently by monolingual and bilingual children. The main difference identified by our study is the absence of indirect WM effects on reading comprehension in bilinguals. This finding, which should be investigated in more depth in future research, suggests that monolingual and bilingual children may differ regarding processing habits and the resources used to implement reading or listening comprehension.

## 6. Limitations

Several limitations of this study must be acknowledged. First, the lockdown in March 2020 due to COVID-19 did not allow us to complete the children’s vocabulary and sentence comprehension assessment. Thus, the analyses carried out in this study cannot clarify whether the indirect WM’s contribution to reading comprehension would remain the same when language skills were considered. A second limitation is that our bilingual group was relatively small, as we selected one school in which the monolingual and bilingual children were likely to have similar socio-cultural backgrounds. Future research should involve a broader sample of monolingual and bilingual children, again similar in terms of the parent’s education, to increase the power of the statistical analyses that have been run.

Finally, the different patterns of association between WM and reading comprehension in monolingual and bilingual children were interpreted, assuming that bilingual children approached text reading and comprehension using more rehearsal processes. These interpretations need to be tested in further research.

## Figures and Tables

**Table 1:** Mean standard scores on working memory and reading measures for monolingual and bilingual children.

	Monolinguals (*n* = 62)		Bilinguals (*n* = 36)		*t* Tests for Independent Samples ^a^
	M (SD)	Range	M (SD)	Range	
Non-word repetition (z scores)	−1.15 (1.56)	−3.1–2.11	−0.99 (1.75)	−3.1–2.11	t(98) = −0.46, *p* = 0.64, d = 0.09
Working Memory Index (standard scores)	99.51 (16.01)	64–148	94 (15.09)	61–127	t(98) = 1.67, *p* = 0.09, d = 0.35
Immediate Narrative Memory (scaled scores)	10.50 (2.69)	2–14	10.25 (2.89)	3–15	t(98) = 0.43, *p* =0.66, d = 0.08
Text reading speed (T scores)	44.40 (7.9)	28–68	42.80 (10.21)	26–71	t(98) = 0.86, *p* = 0.39, d = 0.17
Text reading accuracy (T scores)	44.82 (8.86)	22–65	42.58 (7.18)	26–65	t(98) = 1.28, *p* = 0.20, d = 0.27
Reading comprehension (T scores)	51.93 (8.25)	27–66	47.44 (10.65)	21–66	t(98) = 2.32, *p* = 0.02, d = 0.47
Listening comprehension (T scores)	53.88 (8.10)	36–65	51.94 (7.80)	36–65	t(98) = 1.15, *p* = 0.24, d = 0.24

Note. ^a^ Unadjusted *p*-values are shown. Bonferroni-Holm correction was applied to the pairwise criterion of significance (i.e., alpha levels).

**Table 2:** Correlations between variables for monolinguals and bilinguals.

		Monolinguals (n = 62)	1	2	3	4	5	6	7
	Bilinguals (*n* = 36)								
1	Non-word repetition		-	0.40 **	0.49 **	0.33 *	0.26	0.37 *	0.07
2	WMI		0.21	-	0.28	0.45 **	0.18	0.41 **	0.29
3	Immediate narrative memory		−0.20	−0.06	-	0.39 **	0.23	0.33 *	0.16
4	Text reading speed		0.33 **	0.16	0.10	-	0.17	0.23	0.08
5	Text reading accuracy		0.48 ***	0.30 **	0.07	0.39 **	-	0.41 **	0.25
6	Reading comprehension		0.11	0.21	0.14	0.20	0.24 *	-	0.43 **
7	Listening comprehension		−0.01	0.19	0.50 ***	0.23	0.19	0.24 *	-

Correlations for the monolingual group are shown below the diagonal, and for the bilingual group, above the diagonal. *** *p* < 0.001, ** *p* < 0.01, * *p* < 0.05.

## Data Availability

Data were archived in two hard disks of the Department of Developmental and Social Psychology, Sapienza University of Rome.

## Supplementary Materials

The following supporting information can be downloaded at: https://www.mdpi.com/article/10.3390/brainsci13010058/s1. Table S1: Results of multiple regression analysis using Working Memory Index (WMI) as predictor (Model 1). Table S2: Results of multiple regression analysis using Immediate Narrative Memory (INM) as predictor (Model 2).

## References

[1] Costanzo A., Desimoni M. (2017). Beyond the mean estimate: A quantile regression analysis of inequalities in educational outcomes using INVALSI survey data. Large-Scale Assess. Educ..

[2] INVALSI (2019). Rilevazioni Nazionali Sugli Apprendimenti 2018-19, National Report INVALSI. https://invalsi-areaprove.cineca.it/docs/2019/Rapporto_prove_INVALSI_2019.pdf.

[3] Kintsch W. (1988). The role of knowledge in discourse comprehension: A construction- integration model. Psychol. Rev..

[4] O’Brien E.J., Cook A.E., Lorch R.F. (2015). Inferences during Reading.

[5] Van den Broek P.W., Helder A. (2017). Cognitive processes in discourse comprehension: Passive processes, reader-initiated processes, and evolving mental representations. Discourse Process..

[6] Muijselaar M., Swart N., Steenbeek-Planting E., Droop M., Verhoeven L., de Jong P. (2017). Developmental relations between reading comprehension and reading strategies. Sci. Stud. Read..

[7] Gough P.B., Tunmer W.E. (1986). Decoding, reading, and reading disability. Remedial Spec. Educ..

[8] Hoover W.A., Gough P.B. (1990). The simple view of reading. Read. Writ..

[9] Catts H., Adlof S.M., Ellis Weismer S. (2006). Language Deficits in Poor Comprehenders: A Case for the Simple View of Reading. J. Speech Lang. Hear. Res..

[10] Kendeou P., van Den Broek P., White M.J., Lynch J.S. (2009). Predicting reading comprehension in early elementary school: The independent contributions of oral language and decoding skills. J. Educ. Psychol..

[11] Kim Y.-S.G. (2015). Language and Cognitive Predictors of Text Comprehension: Evidence from Multivariate Analysis. Child Dev..

[12] Tobia V., Bonifacci P. (2015). The simple view of reading in a transparent orthography: The stronger role of oral comprehension. Read. Writ..

[13] Catts H., Hogan T., Adlof S., Catts H., Kamhi A.G. (2005). Developmental changes in reading and reading disabilities. The Connections Between Language and Reading Disabilities.

[14] García J.R., Cain K. (2013). Decoding and reading comprehension: A meta-analysis to identify which reader and assessment characteristics influence the strength of the relationship in English. Rev. Educ. Res..

[15] Goff D., Pratt C., Ong B. (2005). The relations between children’s reading comprehension, working memory, language skills and components of reading decoding in a normal sample. Read. Writ..

[16] Vellutino F.R., Tunmer W.E., Jaccard J.J., Chen R. (2007). Components of reading ability: Multivariate evidence for a convergent skills model of reading development. Sci. Stud. Read..

[17] Florit E., Cain K. (2011). The simple view of reading: Is it valid for different types of alphabetic orthographies?. Educ. Psychol. Rev..

[18] Bellocchi S., Tobia V., Bonifacci P. (2017). Predictors of reading and comprehension abilities in bilingual and monolingual children: A longitudinal study on a transparent language. Read. Writ..

[19] Melby-Lervåg M., Lervåg A. (2014). Reading comprehension and its underlying components in second language learners: A meta-analysis of studies comparing first- and second-language learners. Psychol. Bull..

[20] Babayiğit S., Shapiro L. (2020). Component skills that underpin listening comprehension and reading comprehension in learners with English as first and additional language. J. Res. Read..

[21] Bonifacci P., Tobia V. (2017). The Simple View of Reading in bilingual language-minority children acquiring a highly transparent second language. Sci. Stud. Read..

[22] Kim Y.-S.G. (2016). Direct and mediated effects of language and cognitive skills on comprehension of oral narrative texts (listening comprehension) for children. J. Exp. Child Psychol..

[23] Lervåg A., Aukrust V.G. (2010). Vocabulary knowledge is a critical determinant of the difference in reading comprehension growth between first and second language learners. J. Child Psychol. Psychiatry.

[24] Bonifacci P., Tobia V. (2016). Crossing barriers: Profiles of reading and comprehension skills in early and late bilinguals, poor comprehenders, reading impaired, and typically developing children. Learn. Individ. Differ..

[25] Bonifacci P., Lombardo G., Pedrinazzi J., Terracina F., Palladino P. (2019). Literacy Skills in Bilinguals and Monolinguals with Different SES. Read. Writ. Q..

[26] Baddeley A.D. (2003). Working memory and language: An overview. J. Comm. Disord..

[27] Baddeley A.D., Hitch G., Bower G.H. (1974). Working Memory. The Psychology of Learning and Motivation: Advances in Research and Theory.

[28] Baddeley A. (2000). The episodic buffer: A new component of working memory?. Trends Cogn. Sci..

[29] Jacobson L.A., Koriakin T., Lipkin P., Boada R., Frijters J.C., Lovett M.W., Hill D., Willcutt E., Gottwald S., Wolf M. (2017). Executive Functions Contribute Uniquely to Reading Competence in Minority Youth. J. Learn. Disabil..

[30] Savage R., Lavers N., Pillay V. (2007). Working memory and reading difficulties: What we know and what we don’t know about the relationship. Educ. Psychol. Rev..

[31] Borella E., de Ribaupierre A. (2014). The role of working memory, inhibition, and processing speed in text comprehension in children. Learn. Individ. Diff..

[32] Carretti B., Borella E., Cornoldi C., De Beni R. (2009). Role of working memory in explaining the performance of individuals with specific reading comprehension difficulties: A meta-analysis. Learn. Individ. Diff..

[33] Nouwens S., Groen M.A., Verhoeven L. (2017). How working memory relates to children’s reading comprehension: The importance of domain-specificity in storage and processing. Read. Writ..

[34] Peng P., Barnes M., Wang C., Wang W., Li S., Swanson H.L., Dardick W., Tao S. (2018). A meta-analysis on the relation between reading and working memory. Psychol. Bull..

[35] Swanson H.L., Orosco M.J., Kudo M. (2017). Does Growth in the Executive System of Working Memory Underlie Growth in Literacy for Bilingual Children with and Without Reading Disabilities?. J. Learn. Disabil..

[36] Nouwens S., Groen M.A., Kleemans T., Verhoeven L. (2020). How executive functions contribute to reading comprehension. Bri. J. Educ. Psichol..

[37] Swanson H.L., Sáez L., Gerber M. (2006). Growth in literacy and cognition in bilingual children at risk or not at risk for reading disabilities. J. Educ. Psychol..

[38] Swanson H.L., Sáez L., Gerber M., Leafstedt J. (2004). Literacy and cognitive functioning in bilingual and nonbilingual children at or not at risk for reading disabilities. J. Educ. Psychol..

[39] Raudszus H., Segers E., Verhoeven L. (2018). Lexical quality and executive control predict children’s first and second language reading comprehension. Read. Writ..

[40] Baddeley A.D., Baddeley A.D. (2007). Exploring the episodic buffer. Working Memory, Thought and Action.

[41] Bonifacci P., Tobia V., Lami L., Snowling M.J. (2014). ALCE Assessment di Lettura e Comprensione in Età Evolutiva (Assessment of Reading and Comprehension in Developmental Age).

[42] Gathercole S.E. (1995). Is nonword repetition a test of phonological memory or long-term knowledge? It all depends on the nonwords. Mem. Cognit..

[43] Muter V., Snowling M. (1998). Concurrent and longitudinal predictors of reading: The role of metalinguistic and short-term memory skills. Read. Res. Q..

[44] Snowling M.J., Gallagher A., Frith U. (2003). Family risk of dyslexia is continuous: Individual differences in the precursors of reading skill. Child Dev..

[45] Bertelli B., Bilancia G. (2006). VAUMeLFBatterie per la Valutazione Dell’attenzione Uditiva e Della Memoria di Lavoro Fonologica Nell’età Evolutiva.

[46] Wechsler D. (2003). Wechsler Intelligence Scale for Children-Fourth Edition.

[47] Orsini A., Pezzuti L., Picone L. (2012). WISC-IV Contributo Alla Taratura Italiana.

[48] Nobre A.D.P., Rodrigues J.D.C., Sbicigo J.B., Piccolo L.D.R., Zortea M., Duarte Junior S., Salles J.F.D. (2013). Tasks for assessment of the episodic buffer: A systematic review. Psychol. Neurosci..

[49] Korkman M., Kirk U., Kemp S.L. (2007). NEPSY II. Administrative Manual.

[50] Urgesi C., Campanella F., Fabbro F. (2011). NEPSY II: Contributo Alla Taratura Italiana.

[51] Hayes A.F. (2013). Introduction to Mediation, Moderation, and Conditional Process Analysis.

[52] Holm S. (1979). A simple sequentially rejective multiple test procedure. Scand. J. Stat..

[53] Jeon E.H., Yamashita J. (2014). L2 Reading comprehension and its correlates: A meta-analysis. Lang. Learn..

[54] Kim Y.-S.G. (2017). Why the Simple View of Reading Is Not Simplistic: Unpacking Component Skills of Reading Using a Direct and Indirect Effect Model of Reading (DIER). Sci. Stud. Read..

[55] Graesser A.C., Singer M., Trabasso T. (1994). Constructing inferences during narrative text comprehension. Psychol. Rev..

[56] Jalali-Moghadam N., Kormi-Nouri R. (2017). Bilingualism and reading difficulties: An exploration in episodic and semantic memory. J. Cogn. Psychol..

