# Supplementary material for: How Is Working Memory Related to Reading Comprehension in Italian Monolingual and Bilingual Children?
Source: Brain Sci. 2022 Dec 28;13(1):58. doi: 10.3390/brainsci13010058 (PMC9856821; doi:10.3390/brainsci13010058)
Supplement: Supplementary file 1 [file brainsci-13-00058-s001.zip › brainsci-2090678-supplementary.pdf]

**Table S1.** Results of multiple regression analysis using Working Memory Index (WMI) as predictor (Model 1).

| | ***Estimates*** |
|---|---|
| ***Direct effects*** | |
| WMI → Listening comprehension | 0.17 |
| WMI → Text reading accuracy | 0.28* |
| WMI → Reading comprehension | 0.19 |
| ***Moderator effects*** | |
| Linguistic status → Listening comprehension | −0.23 |
| Linguistic status → Text reading accuracy | −0.20 |
| ***Moderating effects*** | |
| WMI × Linguistic status → Listening comprehension | 0.07 |
| WMI × Linguistic status → Text reading accuracy | −0.16 |
| ***Conditional direct effects*** | |
| WMI → Listening comprehension | |
| Linguistic status 0 (monolingual) | 0.17 |
| Linguistic status 1 (bilingual) | 0.24 |
| WMI → Text reading accuracy | |
| Linguistic status 0 (monolingual) | 0.28* |
| Linguistic status 1 (bilingual) | 0.12 |
| ***Mediators effects*** | |
| Listening comprehension → Reading comprehension | 0.22* |
| Text reading accuracy → Reading comprehension | 0.21* |
| ***Conditional indirect effects*** | |
| WMI → Listening comprehension → Reading comprehension | |
| Linguistic status 0 (monolingual) | 0.04 |
| Linguistic status 1 (bilingual) | 0.05 |
| WMI → Text reading accuracy → Reading comprehension | |
| Linguistic status 0 (monolingual) | 0.06* |
| Linguistic status 1 (bilingual) | 0.03 |
| ***Covariate effects*** | |
| Parents' years of education → Listening comprehension | 0.19 |
| Parents' years of education → Text reading accuracy | 0.12 |
| Parents' years of education → Reading comprehension | 0.10 |

***Notes.*** * $p < .05$; ** $p < .01$. The analyses have been performed by employing Model 7 of the SPSS macro PROCESS (Hayes, 2013). The model included WMI as predictor (X), listening comprehension and text reading accuracy as mediators (M), reading comprehension as criterion (Y), linguistic status (0 = monolingual, 1 = bilingual) as moderator (W) of the relationship between X and M's, and parents' years of education as a covariate.

**Table S2.** Results of multiple regression analysis using Immediate Narrative Memory (INM) as predictor (Model 2).

| | ***Estimates*** |
|---|---|
| ***Direct effects*** | |
| INM → Listening comprehension | 0.51** |
| INM → Text reading accuracy | 0.18 |
| INM → Reading comprehension | 0.09 |
| ***Moderator effects*** | |
| Linguistic status → Listening comprehension | −0.24 |
| Linguistic status → Text reading accuracy | −0.24 |
| ***Moderating effects*** | |
| INM × Linguistic status → Listening comprehension | −0.38* |
| INM × Linguistic status → Text reading accuracy | −0.02 |
| ***Conditional direct effects*** | |
| INM → Listening comprehension | |
| Linguistic status 0 (monolingual) | 0.51** |
| Linguistic status 1 (bilingual) | 0.13 |
| INM → Text reading accuracy | |
| Linguistic status 0 (monolingual) | 0.18 |
| Linguistic status 1 (bilingual) | 0.16 |
| ***Mediators effects*** | |

| | |
|---|---|
| Listening comprehension → Reading comprehension | 0.23* |
| Text reading accuracy → Reading comprehension | 0.24* |
| ***Conditional indirect effects*** | |
| INM → Listening comprehension → Reading comprehension | |
| Linguistic status 0 (monolingual) | 0.12* |
| Linguistic status 1 (bilingual) | 0.03 |
| INM → Text reading accuracy → Reading comprehension | |
| Linguistic status 0 (monolingual) | 0.04 |
| Linguistic status 1 (bilingual) | 0.04 |
| ***Covariate effects*** | |
| Parents' years of education → Listening comprehension | 0.14 |
| Parents' years of education → Text reading accuracy | 0.13 |
| Parents' years of education → Reading comprehension | 0.11 |

***Notes.*** * $p < .05$; ** $p < .01$. The analyses have been performed by employing Model 7 of the SPSS macro PROCESS (Hayes, 2013). The model included INM as predictor (X), listening comprehension and text reading accuracy as mediators (M), reading comprehension as criterion (Y), linguistic status (0 = monolingual, 1 = bilingual) as moderator (W) of the relationship between X and M's, and parents' years of education as a covariat
